# Fundamental Understanding of Oxidative Stability in Fluorinated Asymmetric Ethers for Li Batteries

**DOI:** 10.1002/anie.3143736

**Published:** 2026-06-23

**Authors:** Liang‐Ting Wu, Norio Takenaka, Jyh‐Chiang Jiang, Atsuo Yamada

**Affiliations:** ^1^ Department of Chemical System Engineering School of Engineering The University of Tokyo Bunkyo‐ku Tokyo Japan; ^2^ Department of Chemical Engineering Sustainable Electrochemical Energy Development (SEED) Center National Taiwan University of Science and Technology Taipei Taiwan; ^3^ Research Center For Materials Nanoarchitectonics (MANA) National Institute for Materials Science (NIMS) Tsukuba Ibaraki Japan; ^4^ Sungkyunkwan University Institute of Energy Science and Technology (SIEST) Sungkyunkwan University Suwon‐si Republic of Korea

**Keywords:** density functional theory, fluorinated asymmetric ethers, Li batteries, machine‐learning force field, slow‐growth approaches

## Abstract

Fluorination strategies for electrolyte solvents are widely used to improve the electrochemical performance and cycling stability of Li‐metal batteries. Recently, β‐fluorinated 1‐ethoxy‐2‐methoxyethane (F*x*EME) has been designed and shown to exhibit excellent oxidative stability. However, solvent design remains largely empirical, and its atomistic‐level effects are not fully understood. Here, we investigated how fluorination affects the oxidative stability of asymmetric ethers, (F*x*)EME, using multiscale modeling with density functional theory (DFT), ab initio molecular dynamics (AIMD), and machine‐learning force field (MLFF)‐MD. DFT and AIMD capture fluorination‐induced electronic‐structure changes with high accuracy, while MLFF‐MD enables exploration of interfacial reactions at larger time and length scales. Our results indicate that β‐fluorination stabilizes ethoxy C–H bonds via inductive effects and weakens solvent–cathode (Li_0.5_NiO_2_) interactions through electron redistribution, mitigating oxidation and C–C activation. Furthermore, the increased dipole moment of F3EME drives a preferential orientation that shields the methoxy group. Overall, these three fluorination‐induced effects confer F*x*EME significantly higher oxidative stability than EME. This work provides atomistic insights into fluorination‐driven electronic and interfacial effects, supporting rational electrolyte design.

## Graphical abstract

To improve Li‐metal battery electrolytes, we analyze fluorinated 1‐ethoxy‐2‐methoxyethane (F*x*EME) oxidative stability via density functional theory (DFT) and molecular dynamics (MD). Fluorination strengthens ethoxy C–H bonds and alters solvent orientation at the cathode, weakening interfacial interactions. This suppresses solvent decomposition, providing critical atomistic insights to guide the rational design of stable electrolytes.

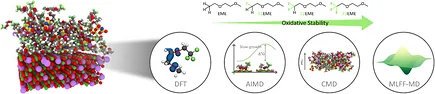

## 1 Introduction

Li‐metal batteries (LMBs) are considered promising energy storage devices with extremely high energy density due to the Li‐metal anode, which features high specific capacity and a low reduction potential [1, 2, 3]. Although the combination of an Li‐metal anode and a high‐nickel cathode can achieve an energy density exceeding 600 Wh kg^−^^1^ at the cell level [4, 5], it suffers from rapid electrolyte degradation at both electrodes [6, 7]. The thickening of the solid electrolyte interphase (SEI) [8, 9, 10, 11] and cathode–electrolyte interphase (CEI) [12, 13] eventually leads to capacity fading and increased interfacial resistance. Therefore, electrolyte engineering strategies, such as developing high‐concentration electrolytes (HCEs) [14, 15, 16], localized high‐concentration electrolytes (LHCEs) [17, 18], high‐entropy electrolytes (HEEs) [19, 20, 21], weakly solvating solvents [22, 23, 24], solvent fluorination [25, 26, 27], and the use of additives [28, 29, 30], are widely adopted to enhance electrochemical performance and cycling stability. However, the overall battery performance is governed by multiple coupled metrics, including oxidative stability, reductive stability, the Li‐ion solvation sheath, ion transport, and Li‐ion desolvation kinetics at interfaces [31, 32, 33, 34]. As a result, electrolyte optimization must consider its influence on all these metrics simultaneously, which poses a significant challenge for rational electrolyte design.

Solvent fluorination strategies have demonstrated effectiveness in enhancing oxidative stability and thereby improving cycling stability [35]. For example, fluoroethylene carbonate (FEC) is a well‐known cosolvent (or additive) that improves interphase quality by forming abundant LiF [36], while methyl (2,2,2‐trifluoroethyl) carbonate (FEMC) exhibits higher oxidative stability on the cathode but decomposes more readily on the anode compared to non‐fluorinated carbonates [37, 38]. Given the better reductive stability of ethers than carbonates, fluorinated ethers are promising for combining an Li‐metal anode with a high‐voltage cathode. Cui et al. reported fluorinated 1,4‐dimethoxybutane (FDMB), 1,5‐dimethoxypentane (FDMP), 1,6‐dimethoxyhexane (FDMH), and 1,8‐dimethoxyoctane (FDMO), where oxidative stability increased with extension of the fluorinated backbone, but salt solubility and ionic conductivity decreased [39]. Bao et al. synthesized a series of β‐fluorinated 1,2‐diethoxyethane (F*x*DEE, *x* = 3, 4, 5, 6) and compared partial and full fluorination at the β‐position of the parent DEE framework [40]. The results indicated that partial fluorination (−CHF_2_) provides higher ionic conductivity, oxidative stability, and Li‐metal Coulombic efficiency than full fluorination [40]. More recently, they further proposed that asymmetric ethers exhibit improved Li‐metal plating–stripping reversibility at high rates and higher exchange current densities than symmetric ethers [41]. The reported fluorinated 1‐ethoxy‐2‐methoxyethane (F*x*EME, *x* = 1, 2, 3) shows higher oxidative stability than EME; in particular, F3EME outperforms the other candidates. Interestingly, the leakage current of Li || NMC811 cells under high voltage was significantly reduced as the degree of fluorination increased [41]. However, fundamental investigations of how fluorination influences the oxidative stability of asymmetric ethers remain limited, and the structure–property relationships in complex systems (i.e., the electrolyte–electrode interface) are still overlooked at this stage.

Benefiting from advances in molecular modeling techniques, detailed electronic structures, intermolecular interactions, and the energy landscape of complex systems can be explored using density functional theory (DFT) calculations [42, 43, 44, 45], ab initio molecular dynamics (AIMD) [46, 47, 48], and machine‐learning force field (MLFF) [49, 50, 51, 52, 53] MD simulations at the molecular and atomistic levels. In this work, we systematically study the oxidative stability of a series of (fluorinated) asymmetric ethers: EME, F1EME, F2EME, and F3EME. We calculated their highest occupied molecular orbital (HOMO) levels, oxidation potentials, and spin densities at the single‐molecule level to compare the effects of fluorination. The solvent–cathode interactions on delithiated lithium nickel oxide (Li_0.5_NiO_2_) were examined through interaction energy and electron‐transfer analyses. In particular, the thermodynamics and kinetics of (F*x*)EME dehydrogenation on the cathode surface were evaluated. In addition, we employed MLFF‐MD simulations to model the dehydrogenation and subsequent decomposition of (F*x*)EME‐LiFSI electrolytes on the Li_0.5_NiO_2_ surface, mimicking realistic interfacial reactions. This work elucidates the effects of fluorination on the electronic properties of ether molecules and solvent–cathode interactions, thereby providing fundamental insights into the mechanisms by which fluorination enhances oxidative stability.

## 2 Results and Discussion

### 2.1 Electronic Structure of (F*x*)EME Solvents

**FIGURE 1:**
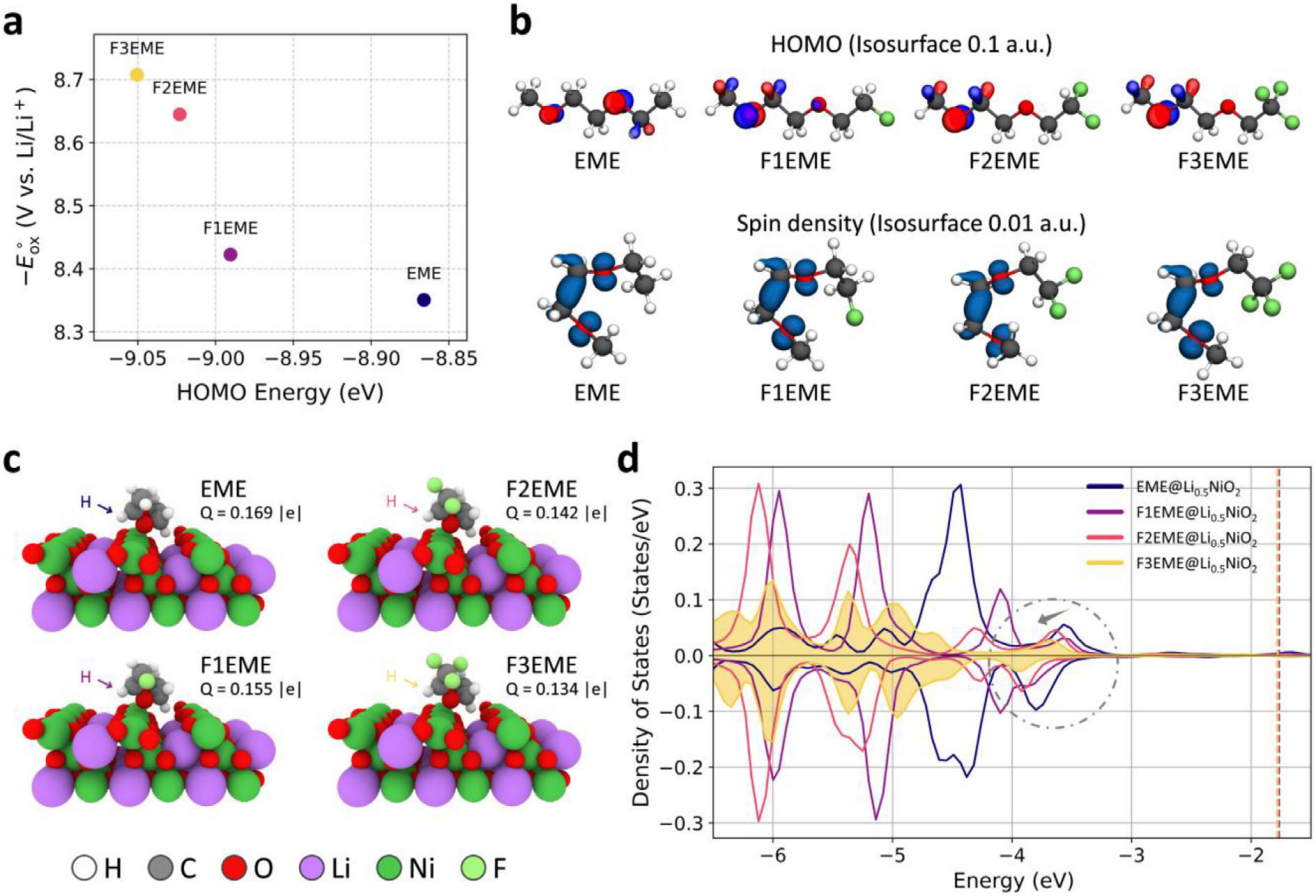
(a) Calculated HOMO energy and oxidation potential of (F*x*)EME solvents. (b) Visualization of the HOMO of neutral solvents and the spin density of oxidized solvents. (c) Optimized geometries of adsorbed (F*x*)EME on the Li_0.5_NiO_2_ surface, with calculated molecular charges (Q) labeled. (d) PDOS of a selected H atom on the ethoxy group in the (F*x*)EME@Li_0.5_NiO_2_ systems. Dashed lines represent the Fermi level of each system.

We calculated the HOMO energy level and the oxidation potential of all (F*x*)EME solvents to investigate their oxidative stability at the molecular level. As shown in Figure 1a, the HOMO energy of F*x*EME decreases as the degree of fluorination (*x*) increases. We visualized the HOMO of (F*x*)EME molecules, as illustrated in Figure 1b. The HOMO of EME exhibits features of non‐bonding electrons from the ethereal oxygen and the C‒H *σ*‐bonding orbitals of the methylene group (‒CH_2_‒) located on the ethoxy group (‒OCH_2_CH_3_). Upon fluorination of the ethoxy group, the HOMO mainly features the C‒H *σ*‐bonding orbitals of the ethylene segment (‒C_2_H_4_‒) and the methoxy group (‒OCH_3_), indicating that the electrons in the C‒H *σ*‐bonding orbitals of the ethoxy group are stabilized by the fluorine. Subsequently, we calculated the oxidation potential of (F*x*)EME and visualized the spin density of the oxidized molecules, [(F*x*)EME]^+^. The spin density indicates the spatial distribution of the removed electron. The oxidative stability ($-E_{ox}^{\circ }$) of (F*x*)EME increases with the degree of fluorination, and the spin density reveals that the electron located in the C‒C *σ*‐bonding orbitals is easily lost after geometric relaxation, weakening the C‒C bond. The weakening of the C–C bond results from the decreased bond order after oxidation. According to the definition, Bond Order = (*n*_bonding_ − *n*_anti‐bonding_)/2, where the bond order is determined by the number of electrons occupying bonding (*n*_bonding_) and anti‐bonding (*n*_anti‐bonding_) orbitals. Therefore, the reduced *n*_bonding_ significantly decreases the bond order and the bond strength. In contrast, the C–O bond, which is generally considered weaker than the C–C bond in a neutral molecule, does not lose electrons in the bonding orbitals in [(F*x*)EME]^+^. Consequently, the C–C bond is expected to play a more significant role in the oxidation process. This trend is general for all [(F*x*)EME]^+^ ions.

Although the HOMO energy and oxidation potential of (F*x*)EME molecules suggest that their oxidative stability improves with increasing fluorination in the ethoxy group, the interfacial reactions between solvents and the cathode surface differ from those of ideal isolated molecules. Therefore, it is necessary to examine the electronic properties of (Fx)EME when adsorbed on the cathode surface. Considering the complicated configuration screening required for commonly used high‐voltage ternary cathodes (LiNi*_x_* Mn*_y_* Co_1‐_ *_x_*_‐_ *_y_* O_2_, or NMC), we simplified the simulation by using the analogue LiNiO_2_. Particularly, LiNiO_2_ is the most representative cathode model because commonly used high‐nickel NMCs (such as NMC‐811 and NMC‐955) are compositionally similar to LiNiO_2_. The electrochemical activity is primarily contributed by Ni and O, rather than Co and Mn. Meanwhile, LiNiO_2_ exhibits high electrochemical reactivity because of the deeply lying 3d band of Ni. This helps in examining the oxidative stability of solvents under more critical conditions. Figure S1 presents the optimized structures of the fully lithiated (LiNiO_2_) and half‐delithiated (Li_0.5_NiO_2_) surface models. We selected the (104) facet as the surface model of LiNiO_2_ because it is the most stable and dominant facet of layered LiNiO_2_, as confirmed computationally and experimentally [54, 55, 56]. Although multiple and mixed exposed facets are more realistic under experimental conditions, choosing the predominant (104) facet is both representative and feasible. First, we calculated the projected density of states (PDOS) of the surface‐exposed nickel and oxygen in LiNiO_2_ and Li_0.5_NiO_2_ (Figure S2). Upon delithiation, their valence bands shift upward and cross the Fermi level, becoming empty states that indicate the partial oxidation of nickel and oxygen. In particular, the near‐Fermi‐level states of oxygen rise after delithiation, providing additional reactivity compared to the initial states. However, this additional reactivity is highly dependent on the local coordination chemistry of oxygen. We compared the PDOS of oxygen atoms with distinct coordination environments, including Li‐coordinated O and non‐Li‐coordinated O, as shown in Figure S3. An intense signal located around 0.2 eV above the Fermi level for non‐Li‐coordinated O suggests a higher oxidation degree than that of Li‐coordinated O, which agrees with chemical intuition. The Li ion is normally trapped by the lattice O when a sufficient electron cloud around the lattice O exhibits O^2−^ features; after partial oxidation of the lattice O, the Li ion leaves the layered structure due to its reduced affinity. Meanwhile, the non‐Li‐coordinated O shows a partial radical feature, where spin‐up and spin‐down signals are located below and above the Fermi level, respectively. The calculated magnetic moments of the surface‐exposed oxygen in LiNiO_2_ and Li_0.5_NiO_2_ are summarized in Figure S4a. Notably, the magnetic moments of several surface oxygen atoms increase from approximately 0.1 *μ*_B_ to 0.4–0.5 *μ*_B_, which clearly reflects their radical character. The intense trap state located 0.2 eV above the Fermi level suggests a strong hydrogen‐abstraction ability of non‐Li‐coordinated O that could trigger proton‐coupled electron transfer (PCET), leading to solvent dehydrogenation. Furthermore, the non‐Li‐coordinated O has a significantly lower steric hindrance for approaching the solvent's hydrogen than the Li‐coordinated O. Therefore, the delithiation process of LiNiO_2_ induces Li vacancies and provides sufficient basicity to the surface non‐Li‐coordinated O.

We examined the electronic properties of (F*x*)EME when adsorbed on Li_0.5_NiO_2_. The optimized geometries are displayed in Figure 1c. The calculated molecular charge of EME is 0.169 |e|, reflecting the amount of electron transfer from the EME molecule to the Li_0.5_NiO_2_ surface after adsorption. The calculated molecular charge decreases progressively with increasing fluorine content, leading to a reduction in the oxidation degree, which implies that the level of C‒C bond activation may differ after fluorination. To further understand the effect of fluorination on solvent–surface interactions, we calculated the interaction energy between the (F*x*)EME solvent and the Li_0.5_NiO_2_ surface, as shown in Figure S5a. The interaction weakens as the degree of fluorination increases, resulting in a longer O_solvent_‒Ni_surface_ distance. The weakening of the interaction is also supported by the reduced accumulation of electron density between the ethereal oxygen and the surface Ni, as shown in Figure S5b. The weakened O‒Ni interaction in the fluorinated solvent can be attributed to the significant downshifting of the valence band of the ethereal oxygen in the ethoxy group (Figure S6a). In comparison, the ethereal oxygen in methoxy shows a similar valence band across different solvents (Figure S6b), which aligns with the HOMO changes observed in pure solvents. Therefore, the weakened O‒Ni interaction mitigates the electron transfer from the solvent to the surface. The calculated PDOS of the selected H atom is shown in Figure 1d. The selected H atoms are located on the methylene group of the ethoxy group and are spatially close to the surface‐exposed oxygen. The PDOS shows that the valence band of the H atom is downshifted and its intensity decreases with increasing degrees of fluorination, reflecting that the oxidation of the selected H in highly fluorinated F*x*EME is more difficult than in EME. This is consistent with the HOMO analysis of the isolated solvents. Additionally, we calculated the PDOS of distinct hydrogen sites on the (F*x*)EME solvent adsorbed on LiNiO_2_ (Figure S7a) and Li_0.5_NiO_2_ (Figure S7b) to examine the spatial range of fluorination‐induced changes in electronic properties. The H atoms located on the ethylene segment (H2 and H3 in Figure S7c) and the methoxy group (H4) did not undergo any significant shift during delithiation compared to the aforementioned ethoxy hydrogen atom (H1). Consequently, we verified that while fluorination can significantly alter local electronic structures, this influence decays rapidly with spatial distance from the fluorination site because the target carbon atoms are separated by at least one ethereal oxygen.

Furthermore, we extended our findings to another commonly used cathode material by evaluating the LiCoO_2_ and Li_0.5_CoO_2_ cathode surfaces (Figure S1). The calculated magnetic moments of the surface oxygen atoms for both models are shown in Figure S4b. The magnetic moments of oxygen on LiCoO_2_ are highly similar to those on LiNiO_2_, featuring a clear O^2−^ character. However, upon delithiation, the magnetic moments of only a portion of the oxygen atoms increased to 0.2–0.35 *μ*_B_. This implies that the basicity of the surface oxygen on Li_0.5_CoO_2_ is significantly lower than that on Li_0.5_NiO_2_. This difference can be attributed to the fact that the 3d bands of Co are shallower (higher in energy) than those of Ni, and are therefore less hybridized with the 2p band of oxygen. We further optimized the geometry of (F*x*)EME adsorbed on the Li_0.5_CoO_2_ surface (Figure S8) and calculated the PDOS of the ethoxy hydrogen atoms (Figure S9). The reduced intensity and downward shift of the valence bands from EME to F3EME confirm that our findings regarding the fluorination effect remain valid when the (F*x*)EME solvent is adsorbed on the Li_0.5_CoO_2_ cathode. Considering that Li_0.5_NiO_2_ presents a more critical condition for solvent dehydrogenation, we mainly focus on Li_0.5_NiO_2_ in the following investigations. Because solid–liquid interfacial systems cannot be described purely by band theory for solids or by molecular orbital theory for isolated molecules, adopting solid‐state concepts such as the Fermi level and valence band can simplify discussion of the PDOS. For clarity, we follow solid‐state terminology in this study. Here, the Fermi level represents the electrochemical potential of electrons, that is, the energy level up to which electronic states are occupied in the system. The valence band refers to the occupied frontier states, which are filled by valence electrons from a specific atom, a group of atoms, or a molecule. These states arise from hybridization between the molecular orbitals of the adsorbed solvents and the electronic bands of the cathode surface.

### 2.2 Thermodynamics of (F*x*)EME Dehydrogenation on the Cathode Surface

**FIGURE 2:**
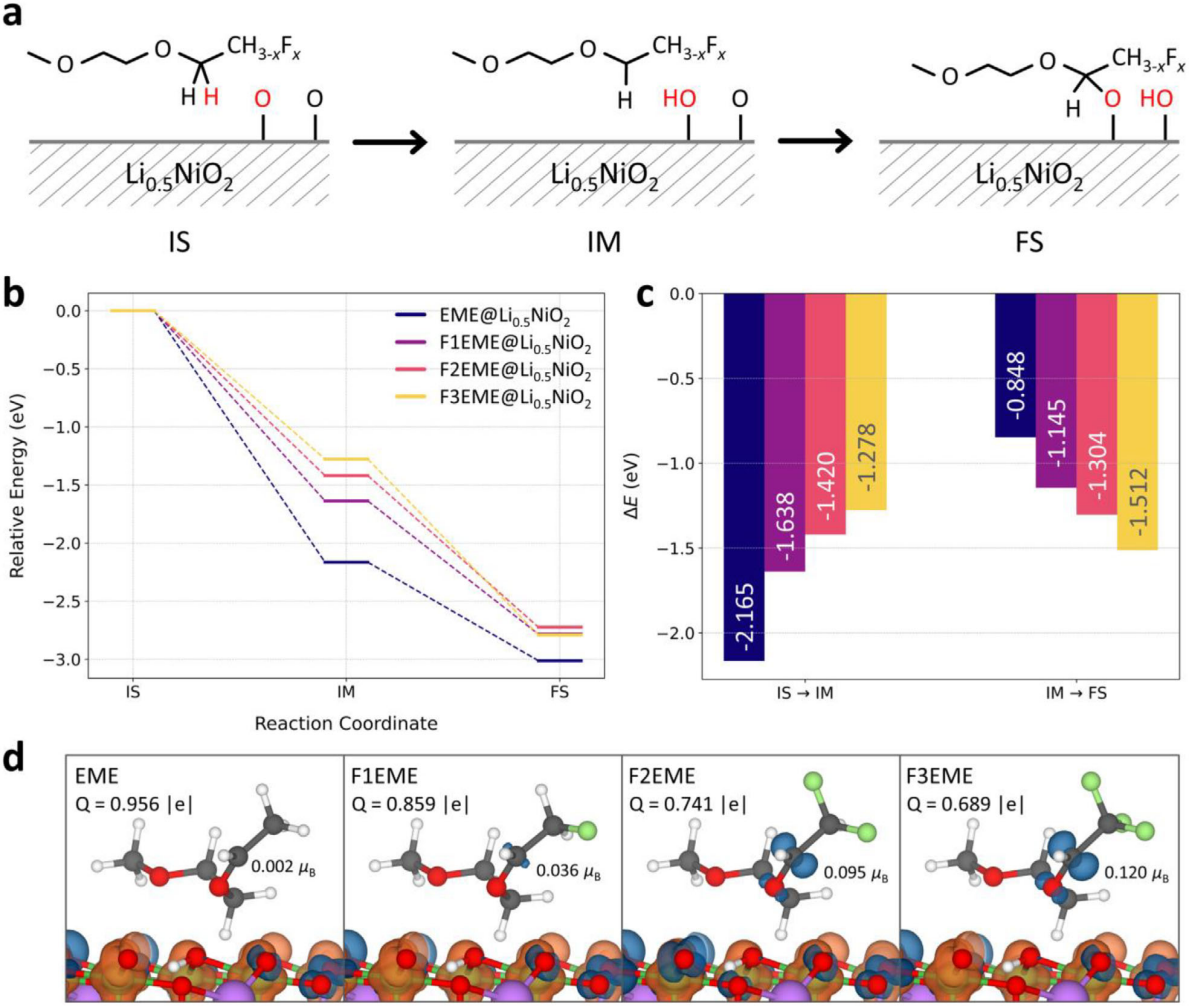
(a) Proposed initial state (IS), intermediate state (IM), and final state (FS) of dehydrogenation of (F*x*)EME solvents on the Li_0.5_NiO_2_ cathode surface. (b) The relative energy of IS, IM, and FS during the dehydrogenation of (F*x*)EME. (c) Energy changes during the IS‐to‐IM and IM‐to‐FS transitions for all (F*x*)EME solvents. (d) Spin density of IM configurations for (F*x*)EME@Li_0.5_NiO_2_ systems, with calculated molecular charges (Q) and the magnetic moment of the *sp* ^2^ carbon labeled.

We further investigated the dehydrogenation of the ethoxy group in adsorbed (F*x*)EME, as presented in Figure 2a. The adsorbed (F*x*)EME (initial state, IS) undergoes C‒H bond cleavage, transferring the H atom to the surface‐exposed oxygen via O‒H bond formation, resulting in the dehydrogenated (F*x*)EME and surface hydroxide (intermediate, IM). The generated dehydrogenated (F*x*)EME then bonds with the surface oxygen through a C‒O coupling reaction, leading to the dissociatively adsorbed (F*x*)EME (final state, FS). We optimized the structures of IS, IM, and FS for all (F*x*)EME and calculated their relative energies (Figure 2b). Both the IS‐to‐IM and IM‐to‐FS reactions for all (F*x*)EME are exothermic, as both surface hydroxide formation and C‒O coupling are highly exothermic processes. Figure 2c compares the energy changes during the IS‐to‐IM (Δ*E*_IS→IM_) and IM‐to‐FS (Δ*E*_IM→FS_) reactions. The former decreases, and the latter increases in magnitude with increasing fluorination. The results indicate that the IM of F*x*EME is less stable than that of EME. For instance, the dehydrogenation of EME is exothermic by 2.165 eV, while it is reduced to only 1.278 eV in F3EME. The instability of the IM in F*x*EME is due to fluorine destabilizing the positive charge generated on the adjacent *sp* ^2^ carbon through the inductive effect of the fluorine substituent. On the other hand, the reaction energy increases with the degree of fluorination during C‒O coupling, as this step converts the unstable IM into a stable coupling species by eliminating the unsaturated *sp* ^2^ carbon.

We calculated the molecular charge of dehydrogenated F*x*EME in the IM configuration, where the dehydrogenated EME carried 0.956 |e|, corresponding to a +1 oxidation state. This value gradually decreases to 0.859, 0.741, and 0.689 |e| for dehydrogenated F1EME, F2EME, and F3EME, respectively. We further visualized the spin density of the IMs (Figure 2d), in which the unpaired electron is distributed on the *sp* ^2^ carbon. The magnetic moment of the carbon increases with higher degrees of fluorination, confirming that more electrons remain on the carbon. This is due to the positive charge being localized on the *sp* ^2^ carbon connected to the highly fluorinated methyl group, making it unstable. The subsequent C‒O coupling is driven by the radical on the *sp* ^2^ carbon. A more pronounced radical feature in the IM configuration is expected to exhibit higher reactivity, which is consistent with the reaction energy analysis. Overall, the thermodynamic profile along the dehydrogenation of (F*x*)EME on the Li_0.5_NiO_2_ cathode surface suggests that the decomposition of F*x*EME can be thermodynamically slowed down by introducing fluorine substituents on the ethoxy group, compared to pristine EME.

### 2.3 Kinetics of (F*x*)EME Dehydrogenation on the Cathode Surface

**FIGURE 3:**
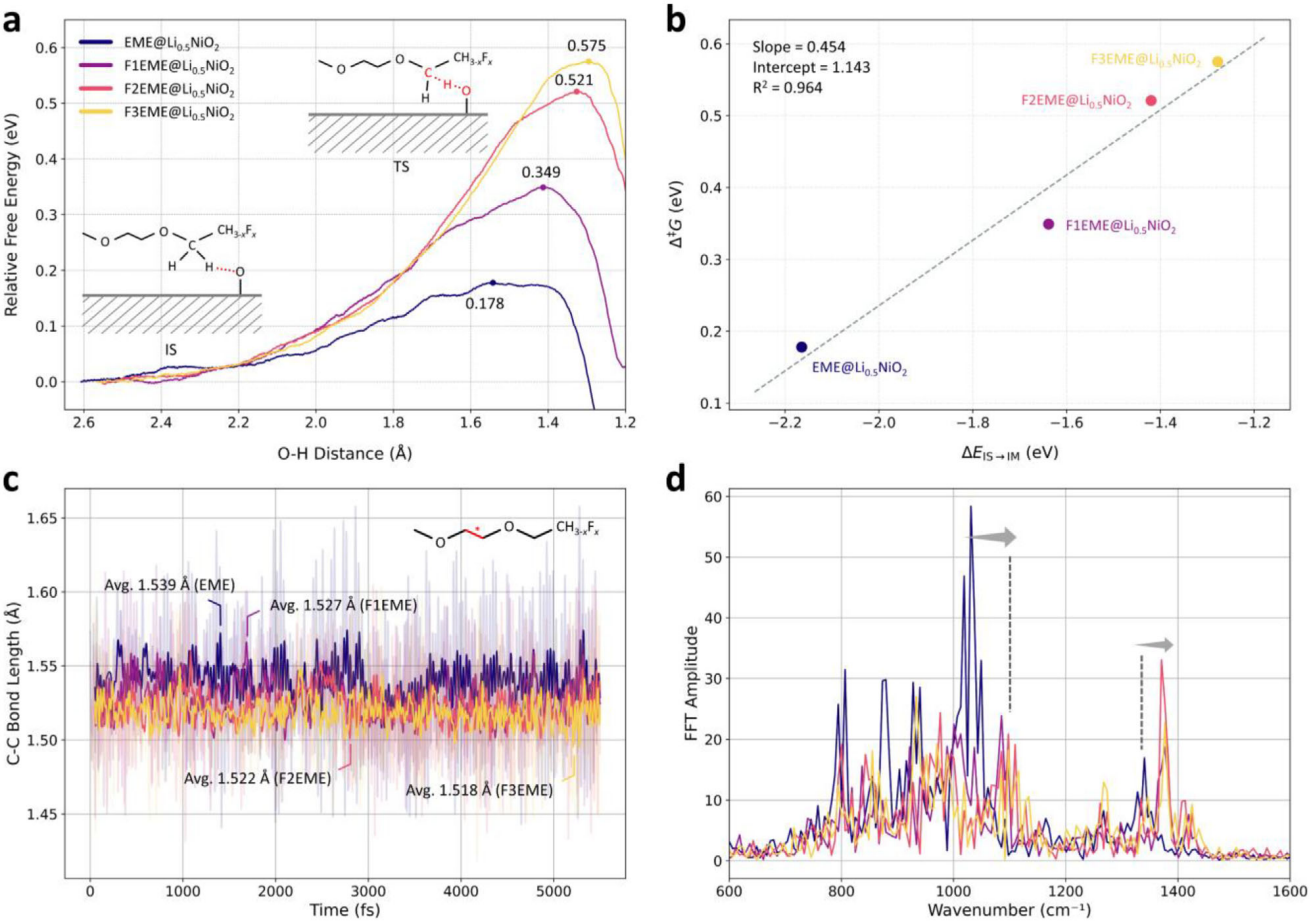
(a) Relative free energy profile along the O‒H distance during the slow‐growth simulation, corresponding to ethoxy group dehydrogenation of the solvents. (b) Correlation between the calculated reaction energy (Δ*E*_IS→IM_) and the activation free energy (Δ^‡^ *G*) for (F*x*)EME dehydrogenation on the Li_0.5_NiO_2_ surface. The gray dashed line represents the linear fit. (c) C‒C bond length fluctuation of the ethylene segment in F*x*EME during the slow‐growth simulation, with the original data faded and the smoothed data highlighted. (d) The fast Fourier transform of the C‒C bond length fluctuation during the simulation.

The kinetics of (F*x*)EME dehydrogenation are crucial for studying solvent stability, as thermodynamics only reveal the reaction tendency when equilibrium is reached, which is insufficient in realistic battery systems. We employed the slow‐growth approach to calculate the free energy profile along the selected geometric coordinate, which captures the potential of mean force (PMF). This method explores the high‐dimensional potential energy surface (PES) along a specific dimension while averaging over the other dimensions. The IS of all (F*x*)EME@Li_0.5_NiO_2_ systems were chosen as the starting point for the slow‐growth simulation, and the distance between the methylene hydrogen and surface oxygen (O‒H distance) was selected as the monitoring geometrical variable, while the other parameters were allowed to evolve freely during the simulation. Although the C–H distance is a more intuitive metric for describing dehydrogenation, it yields unrealistic free energy profiles when the hydrogen atom is not stabilized by surface oxygen, for instance, when the C–H bond points toward the vacuum rather than the surface. Ensuring a specific orientation throughout a simulation is challenging; therefore, while both C–H and O–H distances can theoretically serve as reaction coordinates, the O–H distance is significantly more robust and feasible for capturing the surface‐assisted reaction path. Figure 3a shows the free energy profile along the O‒H distance. The EME system exhibits a maximum free energy of 0.178 eV when the O‒H distance is shortened to 1.543 Å. F1EME has nearly double the barrier at 0.349 eV for a shorter O‒H distance of 1.413 Å compared to EME, and the barrier continues to increase to 0.521 eV for F2EME at an O‐H distance of 1.326 Å. For F3EME, the barrier eventually rises to 0.575 eV, 3.2 times that of EME, with the corresponding O‒H distance reduced to 1.295 Å, only 84% of the value for EME. The simulated results show that the dehydrogenation energy barrier increases significantly with the degree of fluorination and that a shorter O‒H distance is required to trigger hydrogen transfer from the methylene group to surface oxygen. To estimate the effect of the energy barrier on reaction kinetics, we calculated the ratio of the dehydrogenation reaction constant for F3EME to EME using the Eyring equation [57]:

(1) $$\;\frac{k_{F3\mathrm{EME}}}{k_{\mathrm{EME}}}=e^{\frac{\Delta^{‡}G_{\mathrm{EME}}-\Delta^{‡}G_{F3\mathrm{EME}}}{k_{B}T}}\;$$

where *k* represents the rate constant and Δ^‡^ *G* represents the energy barrier for dehydrogenation. The dehydrogenation rate constant for F3EME is only ∼10^−7^ times that of EME. The increase in the energy barrier (activation free energy) aligns with the decrease in the magnitude of the exothermic reaction energy for dehydrogenation, consistent with the Bell–Evans–Polanyi (BEP) principle [58, 59, 60, 61], in which differences in activation energy are proportional to differences in reaction enthalpy for similar reactions. Although we considered only electronic energies rather than enthalpies, the correlation between Δ*E*_IS→IM_ and Δ^‡^ *G* is nearly linear, with an *R* ^2^ value of 0.964. This relationship successfully connects the thermodynamic and kinetic perspectives, as shown in Figure 3b. Notably, the BEP principle is empirical and relies on assumptions, such as the pre‐exponential factors and the position of the transition state (TS) along the reaction coordinate being consistent across all compared reactions. Therefore, it does not exhibit sufficient accuracy for predicting energy barriers based on reaction energy. Nevertheless, it successfully correlates the reaction energy and energy barrier of hydrogenation, and extends the fluorination‐induced destabilization effect on intermediates to the TSs.

We also monitored the C‒H and C‒O distances during the slow‐growth simulation, as shown in Figures S10 and S11, respectively. The dashed lines represent the TS, identified by the maximum free energy. Since the coupling between multiple chemical bond formations and dissociations, we ensured that the subsequent C‒H bond breaking and C‒O coupling occur after the TS. During the slow‐growth simulation, the C‒H distance slightly increases as the O‒H distance decreases, reflecting the weakening of the C‒H bond, which substantially increases after the TS, corresponding to C‒H bond dissociation and hydrogen transfer. Meanwhile, the C‒O distance gradually decreases, suggesting the proximity of the (F*x*)EME fragment to the Li_0.5_NiO_2_ surface. The sudden drop in the C‒O distance after C‒H bond breaking corresponds to the C‒O coupling reaction, where the dehydrogenated (F*x*)EME is anchored on the surface and potentially becomes the initial species for the CEI. Overall, the slow‐growth simulation of (F*x*)EME dehydrogenation suggests that the dehydrogenation of F2EME and F3EME is kinetically unfavorable, with their rate constants being significantly lower than that of EME. In addition, these reactions require a shorter O‒H distance at the TS configurations than in the EME system, as shown in Figure 3a. This implies that fluorination of the ethoxy group can kinetically suppress solvent decomposition on the cathode surface through dehydrogenation of the methylene group. This is consistent with the experimental observations reported by Bao et al. [41], where four electrolytes, consisting of 2 M LiFSI in (F*x*)EME, were tested in Li || NMC811 cells. The constant applied voltage was sequentially fixed at 4.4, 4.5, 4.6, and 4.7 V (vs. Li^+^/Li) for 10 h at each step while the leakage current densities of each cell were monitored. The leakage current represents continuous side reactions, such as the oxidative decomposition of the electrolytes. All electrolytes maintained a low leakage current at 4.4–4.5 V. However, a sudden rise in the leakage current of the EME‐based electrolyte was observed at 4.6 V, suggesting that severe decomposition occurred. The magnitude of the leakage current at each step followed the order of EME > F1EME > F2EME > F3EME‐based electrolytes. Even at 4.7 V, the F3EME‐based electrolyte still maintained a low leakage current, indicating its superior oxidative stability.

Although dehydrogenation on the ethoxy group was confirmed to be suppressed (Figure 3a), dehydrogenation at other potential reaction sites, namely, the hydrogens on the methoxy group and the ethylene segment, warrants examination to identify the spatial limitations of the fluorination effect propagating along the molecule. The relative free energy profiles obtained from slow‐growth simulations for the methoxy group and ethylene segment dehydrogenations of (F*x*)EME are depicted in Figures S12 and S13, respectively. The results show that the magnitude of the energy barrier does not exhibit a clear trend with the degree of fluorination. Meanwhile, all calculated barriers are below 0.45 eV, suggesting highly accessible dehydrogenation pathways via both the methoxy group and the ethylene segment. These simulation results reaffirm that the electronic modifications induced by fluorination are highly localized.

Moreover, the activation of the C‒C bond in the ethylene segment is important for studying the catalytic behavior of the Li_0.5_NiO_2_ cathode surface, as the C‒C bond is closely associated with the oxidation of the (F*x*)EME solvent. We monitored the bond length fluctuation of the C‒C bond during the slow‐growth simulation. The first 500 fs and the frames after 6000 fs were excluded from the analysis to ensure that the properties of the C‒C bond represent the adsorbed solvent state, excluding the states where the temperature had not stabilized by 500 fs and the chemical reactions occurring after 6000 fs. The average C‒C bond lengths of adsorbed EME, F1EME, F2EME, and F3EME were 1.539, 1.527, 1.522, and 1.518 Å, respectively (Figure 3c). Compared with the isolated (F*x*)EME molecules, all molecules exhibit identical C–C bond lengths of 1.514 Å. These results imply that the C‒C bond of EME is activated by the Li_0.5_NiO_2_ surface, which is consistent with the higher oxidation level and stronger O‒Ni interaction of adsorbed EME. The vibrational wavenumbers were obtained through fast Fourier transform (FFT) analysis of the bond length fluctuation, where adsorbed EME exhibited intense signals in the ranges of 1010–1050 and 1310–1360 cm^−1^ (Figure 3d). Fluorination helps maintain the C‒C bond strength, increasing the vibrational wavenumbers to 1080–1120 and 1360–1390 cm^−1^, respectively. As a result, the partial oxidation of the solvent on the Li_0.5_NiO_2_ surface activates the C‒C bond, leading to bond length elongation and a lower vibrational wavenumber. Fluorination of the ethoxy group reduces the oxidation of the solvent on the Li_0.5_NiO_2_ surface, thereby slightly enhancing the C‒C bond strength compared to adsorbed EME.

**SCHEME 1:**
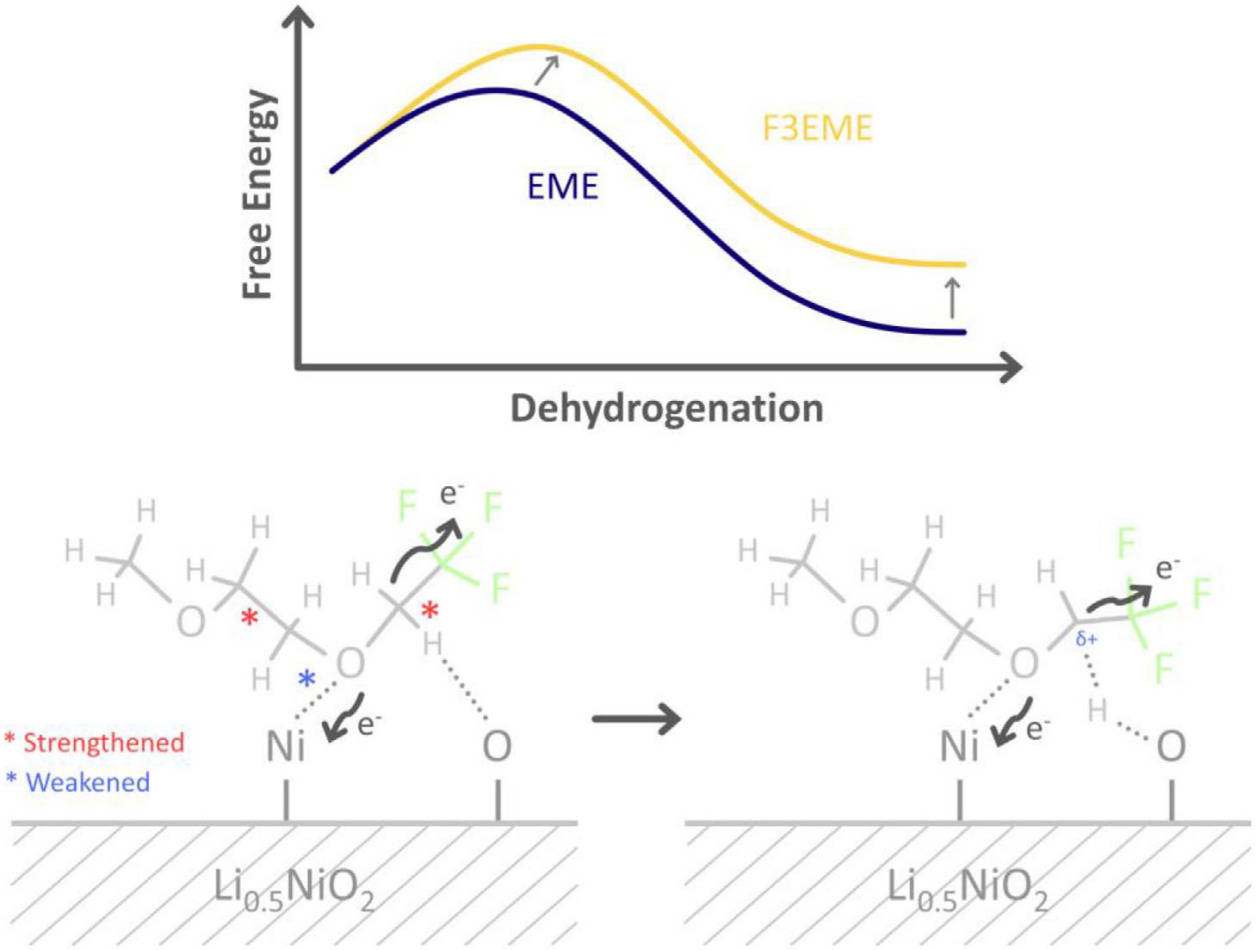
Thermodynamically and kinetically suppressed dehydrogenation of asymmetric ethers through fluorination. The C‒H bond in the ethoxy group and the C‒C bond in the ethylene segment are strengthened, while the O‒Ni interactions are weakened due to fluorination‐induced electron redistribution.

Scheme 1 summarizes the findings of this work, where fluorination of the ethoxy group in EME strengthens the C‒H bond and weakens the O‒Ni interaction through electron redistribution due to withdrawal by the F substituents. The reduced electron transfer to the surface mitigates C‒C activation on the Li_0.5_NiO_2_ cathode surface. Meanwhile, the inductive effect of F destabilizes the *sp* ^2^ carbon generated during dehydrogenation, reducing the reaction energy and increasing the energy barrier, ultimately suppressing dehydrogenation both thermodynamically and kinetically compared to EME. The strengthened C‒C bond in the ethylene group and the C‒H bond in the ethoxy group significantly enhance the oxidative stability of F3EME on the Li_0.5_NiO_2_ cathode.

### 2.4 Large‐Scale MLFF‐MD Simulations of the (F*x*)EME‒Cathode Interface

**FIGURE 4:**
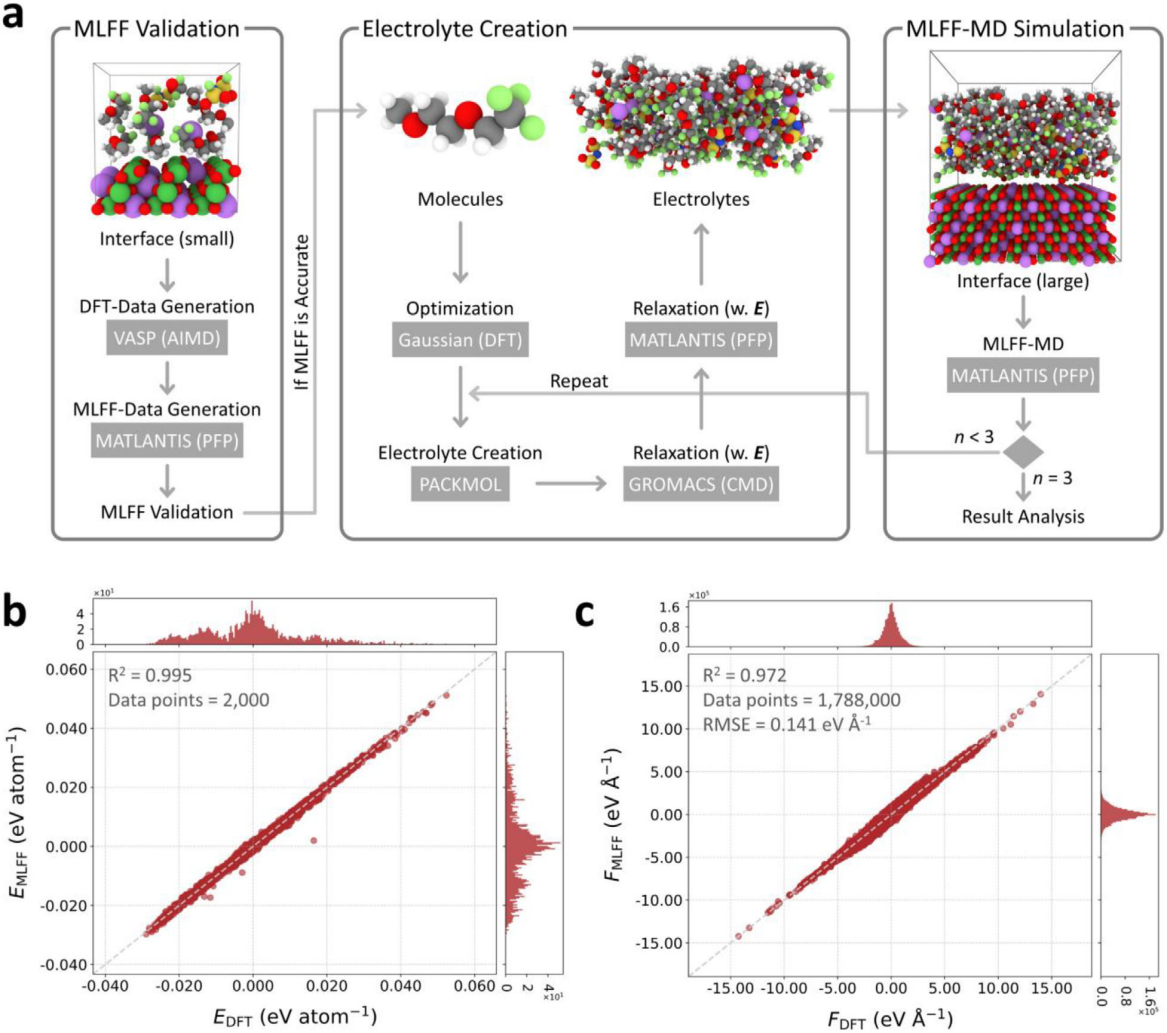
(a) Schematic illustration of the (F*x*)EME:2 M LiFSI@Li_0.5_NiO_2_ interface simulation workflow, built from the molecular level to the large‐scale interfacial model using DFT, CMD, and MLFF‐MD. (b) Comparison of relative DFT‐calculated and MLFF‐predicted energies for 2000 frames generated from AIMD simulations. (c) Comparison of DFT‐calculated and MLFF‐predicted atomic forces for 1 788 000 data points from 2000 frames.

Because the DFT and AIMD results are based on simplified, size‐limited systems with a single solvent molecule on the cathode surface, they are insufficient to capture realistic interfacial reactions. Moreover, (F*x*)EME solvents have multiple reactive sites, including dehydrogenation at the ethoxy group, methoxy group, and ethylene segment, which are not fully covered in the DFT/AIMD studies. Therefore, to further investigate the decomposition of (F*x*)EME solvents on the cathode surface, we performed MLFF‐MD simulations using the pre‐trained preferred potential (PFP) [62, 63], enabling reactive simulations of a larger electrolyte–cathode interface with LiFSI salt and multiple solvent molecules over extended time scales [53]. The workflow, including MLFF validation, electrolyte construction, and MLFF‐MD simulations, is schematically illustrated in Figure 4a. Briefly, we constructed a small interface system and performed short AIMD simulations to generate a set of configurations as the DFT reference dataset. These configurations were then fed into the PFP model to predict system energies and atomic forces. After validating the accuracy of the PFP model against DFT, we constructed large electrolyte systems in which all solvent molecules and the LiFSI salt were first optimized by DFT. Then, four electrolyte systems consisting of the solvents and 2 M LiFSI, as reported in ref. [41], were constructed using PACKMOL (Table S1). The generated electrolytes were subsequently relaxed using a tandem classical molecular dynamics (CMD) and MLFF‐MD procedure. The relaxed electrolyte slabs were then placed on the Li_0.5_NiO_2_ surface to build the large interfacial systems, which served as the initial configurations for long‐term MLFF‐MD simulations. This procedure was repeated three times to obtain statistically meaningful results. Notably, the electrolyte systems were pre‐equilibrated under an applied external electric field, because the interaction between the field and molecular dipole moments is essential for sampling realistic molecular orientations on the surface. Figure S14 shows the electrostatic potential (ESP) surfaces of (F*x*)EME solvents. Introducing fluorine atoms at the terminal causes negative charge accumulation in the ethoxy group, and the dipole moment increases from 0.14 to 2.60, 3.07, and 3.20 Debye upon introducing one, two, and three fluorine atoms, respectively. The larger dipole moment causes the solvent orientation to change near the cathode surface, where the surface carries positive charges under high potential. The fluorinated ethoxy group is expected to orient toward the surface rather than the methoxy group in realistic conditions. Therefore, the stable ethoxy group of highly fluorinated EME preferentially interacts with the surface, reducing the probability of the methoxy group interacting with the surface and increasing the overall stability of F*x*EME molecules, even though the stability of the methoxy group is not enhanced by fluorination.

We constructed a small interface system consisting of 298 atoms and performed AIMD simulations for 3000 fs with a time step of 1 fs. The initial and final configurations are shown in Figure S15. For each interface system, 500 frames were extracted from the 3000‐frame trajectory, and their energies and atomic forces were evaluated using PFP. The energy fluctuations of the four systems are displayed in Figures S16–S19. We subtracted the mean energy of the extracted frames from the absolute energies to focus on relative energy fluctuations. Comparisons of system energies and atomic forces obtained from DFT and MLFF are presented in Figure 4b,c, respectively. The high *R* ^2^ values of 0.995 (energy) and 0.972 (force), together with the low root‐mean‐square error (RMSE) of the atomic forces (0.141 eV Å^−1^), indicate that PFP agrees well with DFT and is reliable for simulating interfacial phenomena. We placed the electrolytes on the surface of a Li_0.5_NiO_2_ cathode with dimensions of 3.62 × 3.43 × 1.41 nm. The electrolyte slabs had the same *a* and *b* lattice parameters as the cathode surface and a thickness of 1.5 nm. The final interface models contained over 3500 atoms, which is ∼12 and ∼25 times more than the systems used to validate PFP and to perform the slow‐growth simulations, respectively. The corresponding configurations are shown in Figures S20–S23. MLFF‐MD simulations were performed for 200 ps to simulate interfacial reactions between the electrolytes and the cathode, which is ∼67 times longer than the AIMD simulations used for validation. We conducted three independent MLFF‐MD simulations because the results can be influenced by different initial configurations and molecular orientations near the surface. Figures S24–S27 show the potential energy fluctuations during the simulations; the energies gradually converge and reach steady states before the end of the runs. Subsequently, we collected all decomposition products formed on the surface for all systems, and the results are summarized in Figures S28–S31.

**FIGURE 5:**
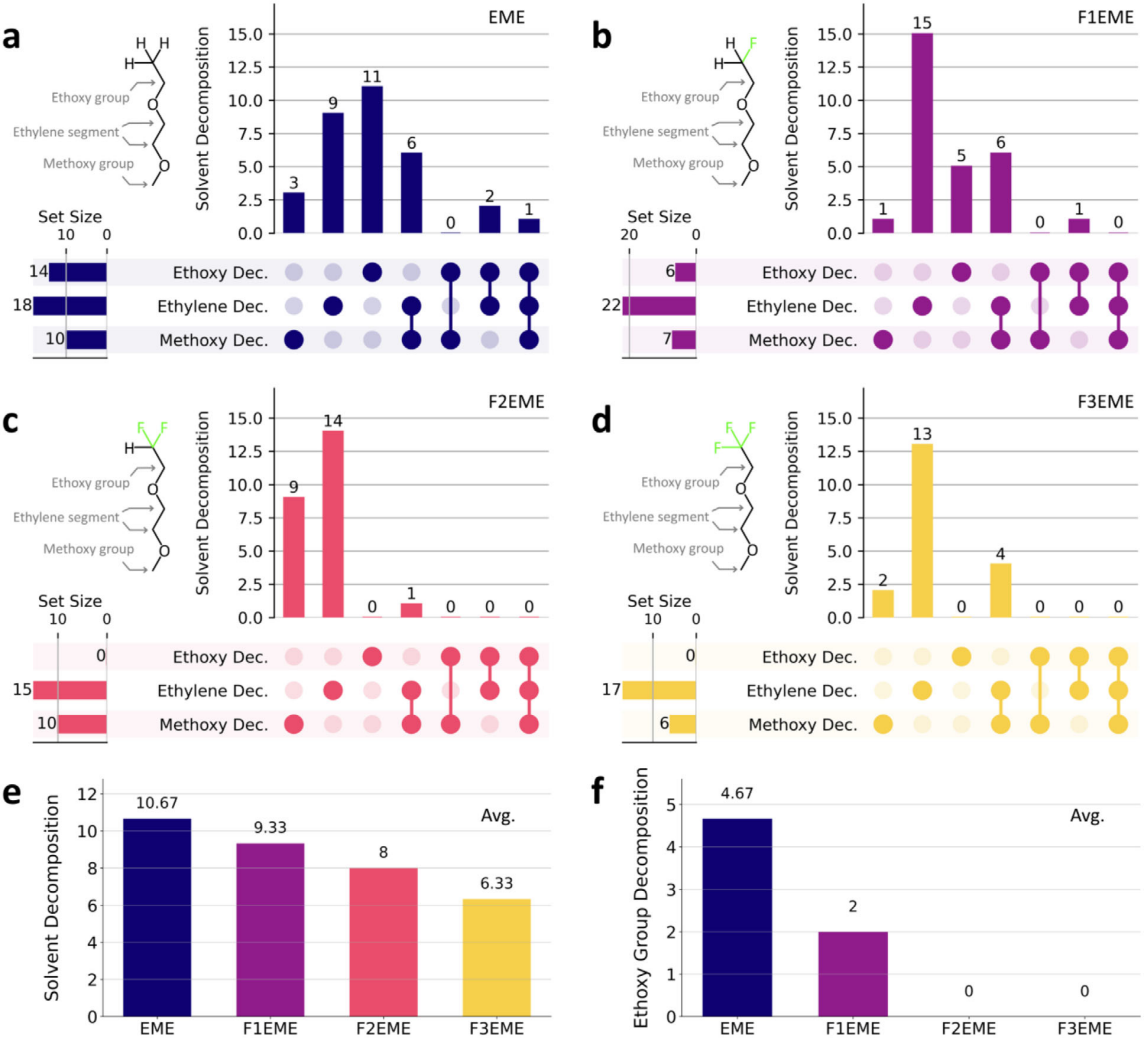
Distribution of solvent decomposition events across indices, including ethoxy group, ethylene segment, and methoxy group decomposition, for (a) EME, (b) F1EME, (c) F2EME, and (d) F3EME:2 M LiFSI electrolyte systems on Li_0.5_NiO_2_. The combinations of decomposed groups in each category are represented by connected solid dots. The number of decomposed solvents in each category and the number involving specific groups are shown by the top bar chart and the horizontal bars on the left, respectively. The decomposition counts are totals collected from three independent MLFF‐MD simulations. (e) Average number of solvent decomposition events and (f) average number of ethoxy group decomposition events across all systems.

Multiple dehydrogenation sites are possible, including the ethylene segment, methoxy, and ethoxy groups, and C–C bond cleavage can also occur. After dehydrogenation, the unsaturated carbon either coordinates with surface oxygen through C–O coupling or triggers a second dehydrogenation on the adjacent carbon to form a C═C bond. We summarized the solvent decomposition events from three independent simulations, as shown in Figure 5a–d. The decomposed solvents were classified into seven distinct categories, including ethoxy group decomposition, ethylene segment decomposition, methoxy group decomposition, decomposition involving any two groups, and decomposition involving all groups. The combinations of decomposed groups in each category are represented by connected solid dots. The number of decomposed solvents in each category and the number involving specific groups are shown by the top bar chart and the horizontal bars on the left, respectively, with values summed over the three simulations. EME did not exhibit a clear preference for dehydrogenation sites: 14, 18, and 10 EME molecules underwent decomposition involving the ethoxy, ethylene, and methoxy groups, respectively. Mono‐fluorination slightly reduced ethoxy‐related decomposition in F1EME from 14 to 6, while the decomposition numbers associated with the ethylene and methoxy groups were similar to those of EME. Di‐fluorination blocked the ethoxy decomposition pathway; the products included 9 methoxy‐only decomposition events, 14 ethylene‐only decomposition events, and 1 event involving both the methoxy and ethylene groups. Similar reactivity was observed for tri‐fluorination, which shows 2 methoxy‐only decomposition events, 13 ethylene‐only decomposition events, and 4 methoxy–ethylene decomposition events. Interestingly, we did not observe methoxy–ethoxy decomposition products for any solvent, likely due to their large spatial separation, and only one EME molecule underwent decomposition involving all groups. In contrast, methoxy–ethylene decomposition products were observed for all solvents, indicating that dehydrogenation at these groups is not blocked by β‐fluorination. This may represent a key target for further improving oxidative stability. The average number of decomposed solvent molecules and the average number of solvents decomposed via ethoxy‐group pathways are summarized in Figure 5e,f, respectively. On average, 10.67 EME molecules decomposed, and 4.67 of these decomposition events involved ethoxy‐group dehydrogenation. Ethoxy‐group dehydrogenation in F1EME is slightly suppressed: the number of decomposed solvent molecules via ethoxy dehydrogenation decreases from 4.67 (EME) to 2 (F1EME), and the average number of decomposed solvent molecules decreases to 9.33 for F1EME. Although the stability of F1EME is slightly improved, ethoxy dehydrogenation remains possible because the calculated reaction barrier of 0.349 eV is not high enough to block this pathway. In contrast, the average number of decomposed solvent molecules in F2EME and F3EME further decreases to 8 and 6.33, respectively. Notably, no ethoxy hydrogens are removed during the simulations for F2EME and F3EME, which is highly consistent with our DFT results, where the energy barriers for ethoxy dehydrogenation increase to 0.521 and 0.575 eV for F2EME and F3EME, respectively. Ethoxy dehydrogenation in F2EME and F3EME may still occur at longer times, at higher delithiation states, or under more extreme charging conditions. Our simulations indicate that the probability of ethoxy dehydrogenation is significantly lower than in EME and F1EME due to both kinetic and thermodynamic suppression. Therefore, F3EME exhibits the highest macroscopic oxidative stability, followed by F2EME, F1EME, and EME, consistent with the DFT results and experimental observations. Furthermore, we did not observe the decomposition of the FSI anion, even though this anion typically contributes to CEI formation, and more complex interactions between the decomposition products of the anion and the solvent might play a crucial role in CEI stability in realistic experiments. Because an applied experimental voltage is absent in current standard AIMD simulations, simulating anion decomposition on the cathode surface is not feasible due to its significantly higher oxidative stability compared to the solvents; consequently, the trained MLFF lacks such data. As a result, our findings highlight the initial solvent decomposition and the enhanced oxidative stability afforded by fluorination. The complete mechanism of CEI formation and anion decomposition must be investigated using improved simulation protocols and specialized MLFFs in the future.

We further performed simulations at the pure solvent–electrode interface to decouple the influence of different solvation structures in electrolyte systems. We fixed the number of solvent molecules at 92 across all systems. The simulation results show that a total of 13 EME, 13 F1EME, 11 F2EME, and 8 F3EME molecules decomposed. Among these decomposed solvents, only 4 EME, 1 F1EME, 2 F2EME, and 0 F3EME followed the ethoxy‐group pathway (Figures S32 and S33). Since the pure solvent system lacks salts that would otherwise partially occupy the surface, the total number of decomposed solvents is higher than that observed in the electrolyte systems. Specifically, the number of decomposed solvent molecules decreased from 13 to 8 after the introduction of three fluorine atoms; furthermore, while 4 EME molecules decomposed via the ethoxy‐group pathway, this number was reduced to 1 and 2 for F1EME and F2EME, respectively, and none of the F3EME molecules decomposed via this route. As a result, the simulations on the pure solvent system support the findings from the previous electrolyte–electrode systems. Notably, the qualitative statistical data from the electrolyte systems remain more reliable than this single‐run test, which merely aims to extend our findings to the pure solvent system.

Interestingly, the experimental data reported in ref. [41] indicated that F3EME exhibits multiple superior properties, including a high exchange current density, excellent ionic conductivity, desirable Li deposition behavior, a highly compact SEI, the lowest amount of dead Li, and enhanced Li plating/stripping reversibility, in addition to the excellent oxidative stability discussed in this work. This outstanding, comprehensive performance of F3EME highlights that the interfacial phenomena between F3EME‐based electrolytes and the Li‐metal surface are also essential, inspiring further studies regarding these metrics in the future. Moreover, a more comprehensive discussion combining oxidative stability with Li‐metal compatibility is expected to advance the rational design of electrolytes in the future.

## 3 Conclusion

In this study, the oxidative stability of the asymmetric ether solvent EME and fluorinated EME (F*x*EME, *x* = 1, 2, 3) is evaluated using DFT calculations, AIMD, and MLFF‐MD simulations. The calculated HOMO shifts from the C–H *σ*‐bonding orbitals on the ethoxy group to those on the methoxy group, accompanied by a downshift in the HOMO level as the degree of fluorination increases. The calculated oxidation potentials of (F*x*)EME show enhanced oxidative stability with increasing fluorination, while the spin density of the oxidized ethers indicates that oxidation is associated with weakening of the C–C bond in the ethylene segment. The strongly electron‐withdrawing F substituents retain more electron density in F*x*EME than in EME on the Li_0.5_NiO_2_ cathode surface. Meanwhile, the downshift of the valence band of the oxygen in the ethoxy group further weakens the O–Ni interaction and mitigates the overall electron transfer from F*x*EME to the surface, thereby reducing C–C bond activation on the catalytic Li_0.5_NiO_2_ surface. Thermodynamic and kinetic analyses of ethoxy‐group dehydrogenation suggest that introducing more F substituents reduces the exothermic reaction energy and increases the energy barrier by destabilizing the intermediate containing a *sp* ^2^ carbon, which is confirmed by spin density and molecular charge analyses of the intermediate configurations. We also performed large‐scale electrolyte–electrode interface simulations using MLFF‐MD to model more realistic conditions, including solvent–solvent interactions and electric‐field‐induced solvent reorientation, with larger interface models and longer simulation times than those accessible by DFT and AIMD. This approach also provides statistically meaningful results by performing repeated simulations with different initial configurations. The simulations show that (F*x*)EME has multiple reactive sites, and dehydrogenation can occur at the ethoxy group, methoxy group, and ethylene segment. Multiple dehydrogenation events on a single solvent molecule were also observed, which can passivate surface oxygen. Regarding ethoxy‐group reactivity, the trend is highly consistent with DFT calculations: the total number of decomposed solvent molecules decreases as the degree of fluorination increases. Notably, the ethoxy dehydrogenation pathway in the F2EME and F3EME electrolytes is completely suppressed in the simulations. Decomposition through the methoxy group and ethylene segment is similar across all solvents, suggesting that the effect of fluorination is mainly localized to the ethoxy group. Nevertheless, the increased dipole moment of F*x*EME with higher fluorination implies that the fluorinated ethoxy group may preferentially orient toward the high‐potential cathode surface, whereas EME shows no clear preference. This agrees with the surface reorientation observed during electrolyte relaxation under an electric field in our MLFF‐MD simulations, which results in the fluorinated ethoxy group pointing toward the cathode surface in the F2EME and F3EME cases. Therefore, the overall oxidative stability increases with the degree of fluorination, even though dehydrogenation via the methoxy group and ethylene segment remains possible. These results highlight that stabilizing these moieties is crucial for further improving solvent oxidative stability. In addition, electric‐field‐induced solvent reorientation is important for suppressing undesired reactive sites and should be carefully considered in molecular design. This work improves fundamental understanding of oxidative stability in fluorinated asymmetric ethers and demonstrates the capability of a multiscale simulation framework combining DFT, AIMD, and MLFF‐MD simulations, thereby supporting future electrolyte design principles.

## Author Contributions

**Liang‐Ting Wu**: conceptualization, investigation, writing – original draft, writing – review and editing, visualization, validation, methodology, software, data curation. **Norio Takenaka**: conceptualization, writing – review and editing, supervision, formal analysis, methodology. **Jyh‐Chiang Jiang**: conceptualization, writing – review and editing, funding acquisition, formal analysis, supervision, project administration. **Atsuo Yamada**: conceptualization, supervision, formal analysis, project administration, writing – review and editing, funding acquisition.

## Conflicts of Interest

The authors declare no conflicts of interest.

## Supporting information



The authors have cited additional references within the Supporting Information [64, 65, 66, 67, 68, 69, 70, 71, 72, 73, 74, 75, 76, 77, 78, 79, 80, 81, 82, 83, 84, 85, 86, 87, 88, 89, 90].
**Supporting File**: anie73312‐sup‐0001‐SuppMat.docx.

## Data Availability

The data that support the findings of this study are available from the corresponding author upon reasonable request.
