# Supplementary material for: Fundamental Understanding of Oxidative Stability in Fluorinated Asymmetric Ethers for Li Batteries
Source: Angew Chem Int Ed Engl. 2026 Jun 23;65(35):e3143736. doi: 10.1002/anie.3143736 (PMC13502641; doi:10.1002/anie.3143736)
Supplement: Supplementary file 1 [file ANIE-65-e3143736-s001.docx]

**Supporting Information**

**Fundamental Understanding of Oxidative Stability in Fluorinated Asymmetric Ethers for Li Batteries**

Liang-Ting Wu,^[a,b]^ Norio Takenaka,*^[a]^ Jyh-Chiang Jiang,*^[b,c]^, and Atsuo Yamada*^[a,d]^

*^a^ Department of Chemical System Engineering, School of Engineering, The University of Tokyo, Hongo 7-3-1, Bunkyo-ku, Tokyo 113-8656, Japan*

*^b^ Department of Chemical Engineering; Sustainable Electrochemical Energy Development (SEED) Center, National Taiwan University of Science and Technology, Taipei 106, Taiwan*

*^c^ Research Center for Materials Nanoarchitectonics (MANA), National Institute for Materials Science (NIMS), Tsukuba, Ibaraki 305-0044, Japan*

*^d^ Sungkyunkwan University Institute of Energy Science and Technology (SIEST), Sungkyunkwan University, Suwon-si 16419, Korea*

**Corresponding Author**

*****Norio Takenaka: [n-takenaka@chemsys.t.u-tokyo.ac.jp](mailto:n-takenaka@chemsys.t.u-tokyo.ac.jp)

*****Jyh-Chiang Jiang: [jcjiang@mail.ntust.edu.tw](mailto:jcjiang@mail.ntust.edu.tw)

*Atsuo Yamada. Email: [yamada@chemsys.t.u-tokyo.ac.jp](mailto:yamada@chemsys.t.u-tokyo.ac.jp)

**Methods**

**Density functional theory (DFT) calculation**

The DFT calculations at the molecular level were performed using the Gaussian 16 package. The Minnesota hybrid meta-generalized gradient approximation functional (meta-GGA), M06-2X,^[64-65]^ was adopted to describe the exchange-correlation interactions of electrons, and the 6-311+G(d,p) basis set^[66-67]^ was used. The SMD implicit solvation model was employed.^[68]^ The oxidation potential was calculated using **Eq. (S1)**,^[69-70]^ where (F*x*)EME and [(F*x*)EME]^+^ were fully optimized and confirmed by all positive vibrational frequencies.

$$E_{ox}^{^{\circ}}=-\frac{G_{\left[ \left( Fx \right)\mathrm{EME} \right]^{+}}-G_{\left( Fx \right)\mathrm{EME}}}{F}-1.37$$

**(S1)**

where the $E_{ox}^{^{\circ}}$ represents the oxidation potential of (F*x*)EME, *G* represents the free energy, and F represents the Faraday constant. The visualization of the highest occupied molecular orbital (HOMO), spin density, and electrostatic potential (ESP) of the molecules was performed using the combined Multiwfn^[71-72]^ and VMD packages.^[73]^

All DFT calculations at the interface were conducted using the Vienna Ab initio Simulation Package (VASP) 6.4.1^[74-75]^ with the projector augmented wave (PAW) method.^[76]^ The Perdew‒Burke‒Ernzerhof (PBE) GGA functional^[77]^ and the DFT‒D3 method^[78]^ were employed to describe the exchange-correlation interactions of electrons and to account for van der Waals interaction corrections. The plane-wave cutoff energy was set to 500 eV, with the convergence threshold for self-consistent field (SCF) iterations set to 10^-4^ eV, and the threshold for ionic relaxation set to 10^-2^ eV Å^-1^. We sampled the Brillouin zone using 3×2×1 Monkhorst–Pack grids^[79]^ for geometry optimization. For the electronic property calculations, such as density of states, a tighter SCF iteration convergence threshold of 10^-5^ eV was used, along with denser Monkhorst–Pack grids of 6×4×1. The Hubbard U value for the Ni 3d orbital was set to 5.96 in the DFT+U framework.^[80-82]^ Atomic charges were calculated using Bader charge analysis.^[83-85]^ The visualization of the spin densities of the IM configuration and the electron density difference after adsorption was performed using the VESTA package.^[71]^ The interaction energy and electron density difference upon adsorption were calculated using **Eqs. (S2)–(S3)**, and the spin density was defined by **Eq. (S4)**, as follows:

$$E_{\mathrm{int}}=E_{\mathrm{ads}}-E_{\mathrm{sur}}-E_{\mathrm{solv}}$$

**(S2)**

$$\Delta\rho=\rho_{\mathrm{ads}}-\rho_{\mathrm{sur}}-\rho_{\mathrm{solv}}$$

**(S3)**

$$\rho_{s}=\rho_{\alpha}-\rho_{\beta}$$

**(S4)**

Here, $E_{\mathrm{int}}$ is the interaction energy, and $E_{\mathrm{ads}}$, $E_{\mathrm{sur}}$, and $E_{\mathrm{solv}}$ are the electronic energies of the adsorbed solvent on the cathode surface, the isolated surface, and the isolated solvent, respectively. $\Delta\rho$ is the electron density difference upon adsorption, and $\rho_{\mathrm{ads}}$, $\rho_{\mathrm{sur}}$, and $\rho_{\mathrm{solv}}$ are the electron densities of the adsorbed solvent on the cathode surface, the isolated surface, and the isolated solvent, respectively. The electronic energies and electron densities of the isolated surface and isolated solvent were also calculated using the same atomic coordinates as in the adsorbed state. $\rho_{s}$ is the spin density, and $\rho_{\alpha}$ and $\rho_{\beta}$ are the electron densities of *α* and *β* electrons, respectively.

**Slow-growth approaches**

The slow-growth approach was employed using DFT within VASP. The Brillouin zone was sampled at the Γ point. The O‒H distance between the surface oxygen and methylene hydrogen was selected as the monitoring geometrical variable, with a transformation velocity of -0.000165 Å step^-1^. The simulation temperature was set to 330 K under the NVT canonical ensemble, where the number of electrons/atoms and the volume were fixed, and the temperature fluctuated around the set value, controlled by the Nosé‒Hoover thermostat.^[86]^ The irreversible work from the initial to an arbitrary state along the selected variable was computed as:^[87-88]^

$$w_{i\to j}^{irrev}=\int_{\xi(i)}^{\xi(j)} \left( \frac{\partial V}{\partial\xi} \right)\cdot\dot{\xi}dt$$

**(S5)**

where $w_{i\to j}^{irrev}$ is the irreversible work from the initial state (*i*) to the arbitrary state (*j*), which is equal to the free-energy difference between the *i* and *j*. $\xi$ is the geometric parameter (i.e., O‒H distance) scanned during the slow-growth simulation, and $\dot{\xi}$ is the transformation velocity of the O‒H distance.

**Machine-learning force field molecular dynamics (MLFF-MD) simulation**

We employed PACKMOL^[89]^ to construct the electrolyte systems ((F*x*)EME-2M LiFSI). The electrolytes were then pre-equilibrated under the NVT ensemble for 1 ns using classical MD simulations (CMD) with the GROMACS package (OPLS-AA force field).^[90]^ Next, the electrolytes underwent an additional 20 ps of pre-equilibration using the preferred potential (PFP) method^[59-60]^ under an applied external electric field of 0.5 V Å^-1^. The electric-field effects considered in CMD and MLFF-MD were implemented by adding an additional electrostatic force term during the simulations, as given in **Eq. (S6)**:

$$\vec{F}_{i,ele}=q_{i}\vec{E}$$

**(S6)**

Here, $\vec{F}_{i,ele}$ is the force on the *i*-th atom induced by the electric field $\vec{E}$, and $q_{i}$ is the atomic charge of the *i*-th atom. The value of $q_{i}$ is specified by the force field in CMD or predicted by PFP in MLFF-MD; the former is fixed, whereas the latter varies with the atomic configuration. The final frame was placed onto the Li_0.5_NiO_2_ surface as the initial structure for large-scale MLFF-MD simulations, which were performed at 330 K under the NVT ensemble for 200 ps.

**Validation of MLFF**

For validation of the PFP model, we constructed small interface systems consisting of eight (F*x*)EME solvent molecules, two LiFSI salts, and a three-layer Li_0.5_NiO_2_ (104) surface with dimensions of 11.38 × 17.29 Å. AIMD simulations were performed under the NVT ensemble for 3,000 fs with a 1 fs time step to generate a set of configurations. The simulation temperature was set to 450 K to accelerate interfacial reactions and sample higher-energy configurations. For each system, we extracted 500 frames from the 3,000-frame AIMD trajectory and evaluated their energies and atomic forces using PFP within MATLANTIS. Overall, we collected 2000 frames across the four (F*x*)EME–LiFSI@Li_0.5_NiO_2_ interface systems, yielding 1,788,000 atomic force data points.

**Interfacial decomposition analysis**

To quantify interfacial degradation, we determined the total number of decomposed solvent molecules on the Li_0.5_NiO_2_ cathode surface at the end of the MLFF-MD simulations based on the final configurations. A chemical bond was identified as broken if its interatomic distance exceeded 2.5 Å, which is a standard threshold for evaluating common bonds such as O–H, C–H, and C–O. Conversely, bond formation was identified if atoms that were initially disconnected exhibited a final interatomic distance shorter than 2.5 Å. The resulting decomposition pathways were classified into seven distinct categories based on combinations of three primary mechanisms: ethoxy group, ethylene segment, and methoxy group decomposition. To quantitatively track this complex reaction system, an indicator function 𝕀(*x*) was introduced to benchmark specific degradation behaviors:

$$\mathbb{I(}x)=\left\{ \begin{aligned} 1 if the solvent is decomposed via pathway x, \\ 0 otherwise \end{aligned} \right.$$

**(S7)**

where *x* represents one of the three pathways. The seven decomposition categories can be expressed as a vector of these three indicator functions: [𝕀(ethoxy), 𝕀(ethylene), 𝕀(methoxy)]. This yields the combinations [1,0,0], [0,1,0], [0,0,1], [1,1,0], [1,0,1], [0,1,1], and [1,1,1], each representing a distinct decomposition product. These results are visualized using an UpSet plot, where the connected solid dots represent the specific indicator vector. The top bar chart displays the number of decomposed solvent molecules corresponding to each category, while the horizontal bars on the left display the total occurrence of each individual decomposition pathway *x* (i.e., the sum of cases where 𝕀(*x*) = 1).

**Table S1.** Numbers of (F*x*)EME solvent molecules, LiFSI salts, and total atoms in the simulation box.

| Systems | Number of solvents | Number of LiFSI salts | Number of atoms |
| --- | --- | --- | --- |
| EME | 92 | 22 | 3732 |
| F1EME | 92 | 22 | 3732 |
| F2EME | 87 | 22 | 3637 |
| F3EME | 81 | 22 | 3523 |

**Figure S1.** Optimized geometries of LiNiO_2_, Li_0.5_NiO_2_, LiCoO_2_, and Li_0.5_CoO_2_ (104) surface models.

**Figure S2.** PDOS of surface-exposed nickel and oxygen on the LiNiO_2_ and Li_0.5_NiO_2_ surfaces. The dashed vertical lines located at 0 eV represent the Fermi level of each system.

**Figure S3.** PDOS of Li-coordinated O and non-Li-coordinated O on the Li_0.5_NiO_2_ surface. The dashed vertical lines located at 0 eV represent the Fermi level of each system.

**Figure S4.** Calculated magnetic moments of surface oxygen atoms in (a) LiNiO_2_ and Li_0.5_NiO_2_, and (b) LiCoO_2_ and Li_0.5_CoO_2_ surfaces.

**Figure S5.** (a) Calculated interaction energy and the O_solvent_‒Ni_surface_ distance for (F*x*)EME adsorbed on the Li_0.5_NiO_2_ surface. (b) Electron density difference between the adsorbed system and the isolated parts, with electron accumulation and depletion corresponding to the increase and decrease in electron density after interaction, respectively.

 **Figure S6.** PDOS of the ethereal oxygen atom on (a) the ethoxy group and (b) the methoxy group in the (F*x*)EME@Li_0.5_NiO_2_ systems. Dashed lines represent the Fermi level of each system.

**Figure S7.** PDOS of H atoms (H1-H4) on (a) EME@Li_0.5_NiO_2_ and (b) F3EME@Li_0.5_NiO_2_ surfaces. The dashed vertical lines located at 0 eV represent the Fermi level of each system. (c) Optimized geometries of adsorbed EME and F3EME on the Li_0.5_NiO_2_ surface, with the ethoxy (H1), ethylene (H2 and H3), and methoxy hydrogen (H4) atoms labeled.

**Figure S8.** Optimized geometries of adsorbed (F*x*)EME on the Li_0.5_CoO_2_ surface, with the ethoxy hydrogen atom labeled.

**Figure S9.** PDOS of a selected H atom on the ethoxy group in the (F*x*)EME@Li_0.5_CoO_2_ systems. Dashed lines represent the Fermi level of each system.

**Figure S10.** Evolution of the C‒H distance along the O‒H distance during the slow-growth simulation. Dashed lines represent the O‒H distance at the maximum free energy value. The significant increase in the C‒H distance reflects the C‒H bond breaking.

**Figure S11.** Evolution of the C‒O distance along the O‒H distance during the slow-growth simulation. Dashed lines represent the O‒H distance at the maximum free energy value. The significant decrease in the C‒O distance reflects the C‒O coupling reaction.

**Figure S12.** Relative free energy profile along the O–H distance during the slow-growth simulation, corresponding to methoxy group dehydrogenation of the solvent.

**Figure S13.** Relative free energy profile along the O–H distance during the slow-growth simulation, corresponding to ethylene segment dehydrogenation of the solvent.

**Figure S14.** Electrostatic potential surfaces (EPS) of (F*x*)EME molecules with the dipole moment labeled.

**Figure S15.** Initial and final configurations from AIMD simulations of the small interface systems used to validate the PFP model, including (F*x*)EME–LiFSI electrolytes on Li_0.5_NiO_2_ cathode surfaces.

**Figure S16.** DFT-calculated and MLFF-predicted energies for 500 frames extracted from a 3000 fs AIMD simulation of the small interface system (EME-LiFSI@Li_0.5_NiO_2_).

**Figure S17.** DFT-calculated and MLFF-predicted energies for 500 frames extracted from a 3000 fs AIMD simulation of the small interface system (F1EME-LiFSI@Li_0.5_NiO_2_).

**Figure S18.** DFT-calculated and MLFF-predicted energies for 500 frames extracted from a 3000 fs AIMD simulation of the small interface system (F2EME-LiFSI@Li_0.5_NiO_2_).

**Figure S19.** DFT-calculated and MLFF-predicted energies for 500 frames extracted from a 3000 fs AIMD simulation of the small interface system (F3EME-LiFSI@Li_0.5_NiO_2_).

**Figure S20.** Initial and final configurations from three independent MLFF-MD simulations of EME:2 M LiFSI electrolytes on Li_0.5_NiO_2_ cathode surfaces.

 **Figure S21.** Initial and final configurations from three independent MLFF-MD simulations of F1EME:2 M LiFSI electrolytes on Li_0.5_NiO_2_ cathode surfaces.

 **Figure S22.** Initial and final configurations from three independent MLFF-MD simulations of F2EME:2 M LiFSI electrolytes on Li_0.5_NiO_2_ cathode surfaces.

**Figure S23.** Initial and final configurations from three independent MLFF-MD simulations of F3EME:2 M LiFSI electrolytes on Li_0.5_NiO_2_ cathode surfaces.

**Figure S24.** Potential energy fluctuations of the EME:2 M LiFSI electrolyte on the Li_0.5_NiO_2_ cathode surface during three independent MLFF-MD simulations.

**Figure S25.** Potential energy fluctuations of the F1EME:2 M LiFSI electrolyte on the Li_0.5_NiO_2_ cathode surface during three independent MLFF-MD simulations.

**Figure S26.** Potential energy fluctuations of the F2EME:2 M LiFSI electrolyte on the Li_0.5_NiO_2_ cathode surface during three independent MLFF-MD simulations.

**Figure S27.** Potential energy fluctuations of the F3EME:2 M LiFSI electrolyte on the Li_0.5_NiO_2_ cathode surface during three independent MLFF-MD simulations.

**Figure S28.** Decomposition products of EME:2 M LiFSI electrolytes on the Li_0.5_NiO_2_ cathode surface after 200 ps MLFF-MD simulations. Compounds shown in blue and black are produced via the ethoxy decomposition pathway and other pathways, respectively.

**Figure S29.** Decomposition products of F1EME:2 M LiFSI electrolytes on the Li_0.5_NiO_2_ cathode surface after 200 ps MLFF-MD simulations. Compounds shown in blue and black are produced via the ethoxy decomposition pathway and other pathways, respectively.

**Figure S30.** Decomposition products of F2EME:2 M LiFSI electrolytes on the Li_0.5_NiO_2_ cathode surface after 200 ps MLFF-MD simulations. Compounds shown in blue and black are produced via the ethoxy decomposition pathway and other pathways, respectively.

**Figure S31.** Decomposition products of F3EME:2 M LiFSI electrolytes on the Li_0.5_NiO_2_ cathode surface after 200 ps MLFF-MD simulations. Compounds shown in blue and black are produced via the ethoxy decomposition pathway and other pathways, respectively.

**Figure S32.** Decomposition products of (F*x*)EME on the Li_0.5_NiO_2_ cathode surface after 200 ps MLFF-MD simulations. Compounds shown in blue and black are produced via the ethoxy decomposition pathway and other pathways, respectively.

**Figure S33.** (a) Number of solvent decomposition events and (b) average number of ethoxy group decomposition events across all pure solvent systems.
